# Chirality in Atomically Thin CdSe Nanoplatelets Capped with Thiol-Free Amino Acid Ligands: Circular Dichroism vs. Carboxylate Group Coordination

**DOI:** 10.3390/ma17010237

**Published:** 2024-01-01

**Authors:** Daria A. Kurtina, Vladimir B. Zaytsev, Roman B. Vasiliev

**Affiliations:** 1 Department of Chemistry, Lomonosov Moscow State University, 119991 Moscow, Russia; dashutakarlova@mail.ru; 2 Department of Physics, Lomonosov Moscow State University, 119991 Moscow, Russia; vzaitsev@phys.msu.ru; 3 Department of Materials Science, Lomonosov Moscow State University, 119991 Moscow, Russia

**Keywords:** 2D semiconductors, chirality, CdSe nanoplatelets, circular dichroism, amino acids, excitons, ligand exchange, colloidal synthesis

## Abstract

Chiral semiconductor nanostructures and nanoparticles are promising materials for applications in biological sensing, enantioselective separation, photonics, and spin-polarized devices. Here, we studied the induction of chirality in atomically thin only two-monolayer-thick CdSe nanoplatelets (NPLs) grown using a colloidal method and exchanged with L-alanine and L-phenylalanine as model thiol-free chiral ligands. We have developed a novel two-step approach to completely exchange the native oleic acid ligands for chiral amino acids at the basal planes of NPLs. We performed an analysis of the optical and chiroptical properties of the chiral CdSe nanoplatelets with amino acids, which was supplemented by an analysis of the composition and coordination of ligands. After the exchange, the nanoplatelets retained heavy-hole, light-hole, and spin-orbit split-off exciton absorbance and bright heavy-hole exciton luminescence. Capping with thiol-free enantiomer amino acid ligands induced the pronounced chirality of excitons in the nanoplatelets, as proven by circular dichroism spectroscopy, with a high dissymmetry g-factor of up to 3.4 × 10^−3^ achieved for heavy-hole excitons in the case of L-phenylalanine.

## 1. Introduction

Chiral semiconductor nanostructures and nanoparticles have attracted tremendous interest due to a variety of intriguing properties [1,2,3]. Due to the chiral shapes of the nanostructures and/or the chirality of the molecular orbitals forming excited states in semiconductor nanostructures, excitons acquire mirror asymmetry, which leads to different interaction with left- and right-handed circularly polarized photons [4]. Such nanostructures with chiral excitation are promising candidates for biosensing, stereoselective reactions, and enantioselective separation [5,6,7]. Moreover, a manifestation of chirality in solid state is the chirality-induced spin selectivity effect [8], which leads to the spin polarization of electrons scattered on a dissymmetric center, which opens up new prospects for creating spintronics devices [9,10].

One of the most common approaches to the synthesis of chiral semiconductor nanostructures and nanoparticles is the attachment of enantiomeric ligands to the semiconductor core of the nanoparticle [11,12,13,14,15,16], which results in the appearance of circular dichroism (CD), the difference in the absorption of left- and right-handed circularly polarized light. Among other nanoparticles, a new class of colloidal semiconductor nanostructures comprises cadmium chalcogenide nanoplatelets (NPLs) with a 2D electronic structure [17]. Due to quantum confinement in one dimension and atomically precise thickness, these NPLs demonstrate pronounced and record narrow excitonic bands among other nanoparticles, which promises applications in light-emitting devices [18,19], lasers [20,21], and white-light generation [22,23]. Pronounced circular dichroism of excitonic bands was shown for colloidal CdSe NPLs capped with enantiomeric ligands [24,25]. For atomically thin CdSe NPLs with an only two-monolayer (ML) thickness, record values of circular dichroism have been demonstrated, exceeding those for other types of semiconductor nanoparticles, which is due to the maximum interaction of excitons in the 2D core of the NPL and chiral ligands [26].

Regarding the mechanism of ligand-induced chirality, the nature of the chirality of semiconductor nanostructures and nanoparticles induced by ligands is still a hotly debated topic. There are several formulated theories, including the direct interaction of the energy levels of a semiconductor and an optically active molecule with subsequent hybridization [27], the interaction of moments of electric dipole transitions of chromophores [25], chiral distortions of the nano-crystal lattice [28], and chiral defects in particular [12,29]. Thus, to induce chiral excitons, the coordination of ligands on the nanoparticle surface plays an important role [30]. It was shown that L/D cysteine and N-acetylcysteine ligands induce different signs of circular dichroism at the same absolute configuration for both 0D CdSe quantum dots and 2D CdSe nanoplatelets [31,32]. The change in the sign of the CD is due to a change in ligand coordination when replacing cysteine with acetylcysteine, leading to a change in the orientation of the molecular dipole and the exciton dipole in the core. Also, different signs of circular dichroism were shown for L/D of cysteine on CdSe nanoplatelets with different crystal structures of wurtzite and zinc blende due to differences in ligand coordination [24]. However, almost all studies on the induction of chirality in nanoparticles have concerned thiol-containing enantiomeric ligands, usually thiolated amino acids (cysteine and its derivatives), which are not always the best ligands for practical applications, since the thiolate group quenches luminescence [33,34,35]. Research on thiol-free ligands is quite limited; there are data on the use of chiral carboxylic acids coordinated by the carboxyl group on the surface of CdSe quantum dots, which enhances circular dichroism [36].

**Figure 1:**
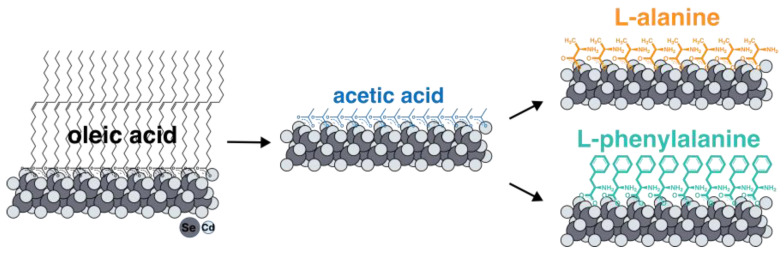
The scheme of a two-step ligand exchange technique with intermediate substitution for short-chain acetic acid. Intermediate substitution with acetic acid is thermodynamically advantageous due to reduced solubility in a non-polar solvent and precipitation of NPLs. Further substitution for target amino acids ligands occurs at elevated temperatures with the distillation of acetic acid.

Here, we report a study of the induction of chirality using thiol-free ligands on the surfaces of atomically thin CdSe nanoplatelets. We chose the model thiol-free amino acids L-alanine and L-phenylalanine as thiol-free ligands. We analyzed ultimately thin 2 ML thick (thicknesses of 0.6 nm) nanoplatelets grown using the colloidal method to maximize the influence of the ligand on induced chirality and achieve strong circular dichroism. We have developed a novel two-step approach (Figure 1) to completely exchange the native oleic acid ligand for chiral amino acids at the basal planes of NPLs. We analyzed the composition and coordination of ligands in detail using Fourier-transform infrared spectroscopy (FTIR). The optical and chiroptical properties of chiral atomically thin CdSe NPLs have been studied via absorption, luminescence, luminescence excitation, and circular dichroism (CD) spectroscopy.

## 2. Materials and Methods

Cadmium acetate dihydrate (Cd(CH_3_COO)_2_∙2H_2_O, ≥98%), selenium powder (Se, 99.99%), trioctylphosphine (TOP, 90%), oleic acid (OA, 90%), 1-octadecene (ODE, 90%), L-alanine (Ala, ≥98%), L-phenylalanine (Phe, ≥98%), acetic acid (AcA, ≥99.7%), and solvents were purchased from Sigma-Aldrich. The cadmium acetate dihydrate used had previously been recrystallized to avoid hydrolysis.

The growth of the initial two-dimensional 2.5 ML thick CdSe nanoparticles coated with oleic acid (CdSe394OA) was carried out through the colloidal method in the octadecene–cadmium acetate–oleic acid system according to the method adapted from [26]. Shortly, 160 μL of oleic acid and 0.26 g of freshly recrystallized cadmium acetate dihydrate were added to 20 mL of octadecene and the resulting mixture was degassed under argon flow for 45 min at a temperature of 160–170 °C. After that, the mixture was cooled to the injection temperature (120 °C), and under vigorous stirring, 0.2 mL of 1 M Se solution in TOP diluted to 0.5 mL with ODE was injected rapidly into the reaction mixture, initiating nanosheet nucleation. The growing of nanoparticles was carried out for 5 h, gradually increasing the temperature to 140 °C. To complete the synthesis, 2 mL of oleic acid was injected and the resulting solution was cooled to room temperature. The final nanoparticles were precipitated through centrifugation with acetone as a precipitator at 5000 rpm for 10 min. Finally, the sample was dissolved in 2 mL of hexane.

Ligand exchange with L-alanine (Ala) and L-phenylalanine (Phe) ligands was carried out according to the newly introduced method with an intermediate substitution for acetic acid. Briefly, 500 μL of acetic acid was added to a solution of 2 mL of the original CdSe394OA nanoparticles coated with oleic acid in 6 mL of hexane, after which the mixture was intensively mixed and left at room temperature for 24 h. The resulting precipitate (CdSe394AcA) was centrifuged, after which it was washed with pure hexane. Centrifugation was repeated several times to wash off the remnants of oleic acid. The resulting nanoparticles were redispersed in 2 mL of hexane. Further exchange for L-alanine and L-phenylalanine was carried out in dioxane. For this purpose, the solvent was previously degassed. To 10 mL of dioxane in the flask, 2 mL of a solution of nanoparticles in hexane and 50 mg of acid were added. The exchange, in which the distillation of acetic acid took place, was carried out for 40 min at 80 degrees. The resulting nanoparticles were precipitated using centrifugation with acetone as a precipitator. The final samples named CdSe394Ala and CdSe394Phe were dispersed in 1 mL of toluene.

Transmission electron microscopy (TEM) images were obtained on the LEO19 AB OMEGA microscope operated at 100 kV. Fourier-transform infrared spectroscopy (FTIR) spectra were registered on a Perkin-Elmer Frontier FTIR spectrometer in the 400–4000 cm^−1^ wavenumber range at room temperature. Samples for analysis were prepared by mixing a drop of NPLs solution or reference Ala or Phe solution in toluene with KBr powder followed by pressing into tablets after solvent evaporation. Absorption spectroscopy was carried out in the 200–800 nm wavelength range with scanning speed of 100 nm/min on the Carry50 (Varian) spectrophotometer. Photoluminescence spectra were collected with a LS 55 (Perkin-Elmer) fluorescence spectrometer. Circular dichroism (CD) spectra were recorded on spectropolarimeter Chirascan (Applied Photophysics) in 300–500 nm wavelength range with scanning speed of 10 nm/min and 1 nm step (integration time 3 s). Optical measurements were carried out at room temperature from colloidal solutions of NPLs in toluene or methanol diluted to an optical density of <1. Quartz cuvettes (Hellma Analytics) with 0.2 cm optical path length were used. Dissymmetry g-factor was calculated as g = ΔA/A = (A_L_ − A_R_)/A, where A_L_ and A_R_ are the absorbances of circularly polarized left-handed and right-handed light, respectively and A is the absorbance of unpolarized light.

## 3. Results

### 3.1. Atomically Thin Nanoplatelet Growth and Ligand Exchange

**Figure 2:**
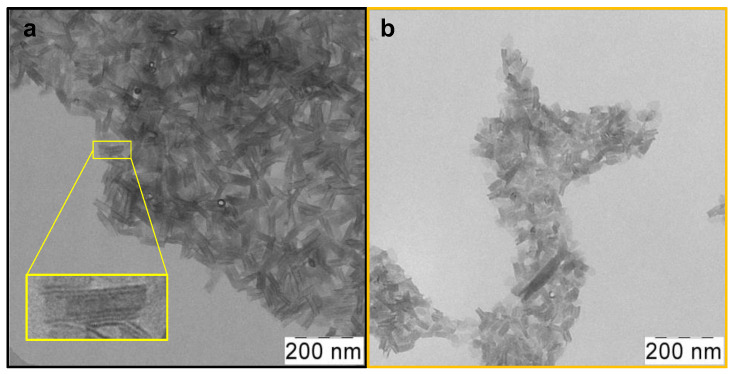
Low-magnification TEM images of (**a**) as-synthesized scroll-like CdSe394OA NPLs covered with oleic acid ligands and (**b**) the same NPLs after ligand exchange with L-Ala. The insert in yellow frame shows a scroll-like form of NPL.

Atomically thin CdSe 2.5 ML NPLs were synthesized using the colloidal growth method. To obtain oleic acid (OA)-covered CdSe nanoparticles with zinc blende crystal structure and ultimately thin thickness, a modification of the standard synthesis protocol [26] was made for the case of low temperatures. As a result, CdSe NPLs with a thickness of 2.5 ML or 0.6 nm were obtained. Their lower-energy exciton transitions occur at a wavelength of 394 nm, so we denoted them CdSe394OA. According to TEM, nanoparticles that were sufficiently uniform in shape and size as-synthesized were obtained (Figure 2a). One can see an ensemble of nanoplatelets, which roll into nanoscrolls due to the spontaneous folding effect [37]. The average length of such scrolls, in this case, is about 80 nm.

To exchange the ligands of native oleic acid on the surfaces of nanoparticles for amino acids, it turned out to be necessary to develop a new technique. All the approaches described in the literature for ligand replacement on the basal planes of CdSe NPLs suggest the substitution of the carboxylate group of oleic acid with the sulfhydryl group of cysteine or another thiol-containing ligand, which has a greater affinity for cadmium surface cation [38]. It easily can be realized with the use of the phase-transfer method and organic solvent method [26,39]. At the same time, substitution for amino acid ligands involves replacing one carboxyl group with another, which means it is not feasible according to the earlier-reported synthesis protocols. For such an exchange, we developed, for the first time, a new technique with intermediate substitution for short-chain acetic acid (Figure 1). Such substitution is thermodynamically advantageous and occurs at room temperature due to the precipitation of nanoparticles as a result of a gradual decrease in solubility in non-polar solvents when the surface is coated with acetic acid. The short-chain acetic ligand was then replaced with the target amino acids at elevated temperature, removing free acetic acid via distillation to shift the equilibrium. The completeness of such an exchange with an intermediate stage of substitution for acetic acid was confirmed by the FTIR spectra (Figure S1).

After the exchange of oleic acid on the surfaces of nanoparticles for the alanine ligand (Figure 2b), the scroll-like morphology is preserved with some loss of uniformity. This may be due to the multistep nature of the exchange process and requires further optimization of synthesis conditions. Meanwhile, after exchange with the phenylalanine ligands, the NPLs unfold (Figure S2). This may indicate both the volume of the substituent and a more complete exchange. The size distribution of nanoparticles for both ligands is shown in Figure S3.

### 3.2. FTIR Analysis of Ligand Coordination

**Figure 3:**
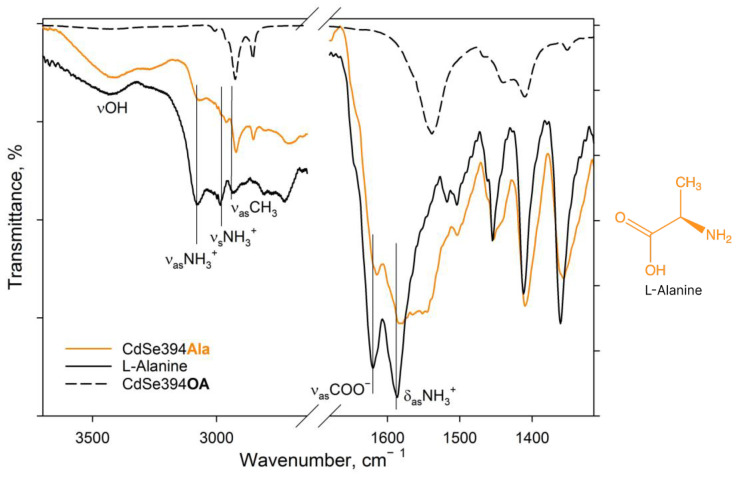
FTIR spectra of L-Ala-capped CdSe394Ala (orange line) sample. Black solid line shows the FTIR spectrum of free L-alanine reference. Dashed black line shows the FTIR spectrum of the initial oleic-acid-capped CdSe394OA sample. Positions of the main vibration bands are marked by black solid lines. The spectra are offset for clarity.

The completeness of amino acid ligand substitution on the basal surfaces of CdSe nanoparticles and their coordination on them were analyzed using the FTIR method. Figure 3 shows the FTIR spectra for pure L-Alanine, a sample coated with the L-Alanine ligand, and an initial sample with oleic acid on the surface.

In the spectrum of pure alanine (Figure 3, black solid line), a band of about 3440 cm^−1^ refers to -OH stretching vibrations of the hydroxyl group, which can occur both from the partially uncharged state of the alanine molecule and from water absorbed by the tablet from the air during measurements. The 3290 cm^−1^ band is attributed to valence vibrations of the NH_2_ group, the presence of which also indicates the presence of a partially uncharged state of the L-Alanine molecule [40]. But, the pronounced bands at 3087 and 3000 cm^−1^ are attributed in the literature to asymmetric and symmetrical oscillations of the charged NH_3_^+^ group in the zwitterionic state [40,41,42]. The charged state is indicated by the presence of a band of valence-asymmetric COO− vibrations at 1620 cm^−1^ and deformation-asymmetric NH_3_^+^ vibrations at 1587 cm^−1^ [40,41,43]. In the C-H deformation region, asymmetric (1455 cm^−1^) and symmetric (1410 cm^−1^) deformation vibrational modes of CH_3_ are defined.

When switching to the CdSe394Ala sample capped with the L-Alanine ligand on the surface spectrum (Figure 3, orange line), one can observe the preservation of the main vibrational band’s characteristic of L-Alanine, but with a shift in their position and broadening of the bands. Thus, it is possible to observe the broadening of the band of asymmetric vibrations of the carboxylate group as a result of its attachment to the surface of nanoparticles. It is also worth noting the presence of a minor contribution of vibrational bands from unwashed oleic acid on the surface when compared with CdSe394OA, the presence of which is also visible at the previous stage of exchange with acetic acid (see Figure S1).

**Figure 4:**
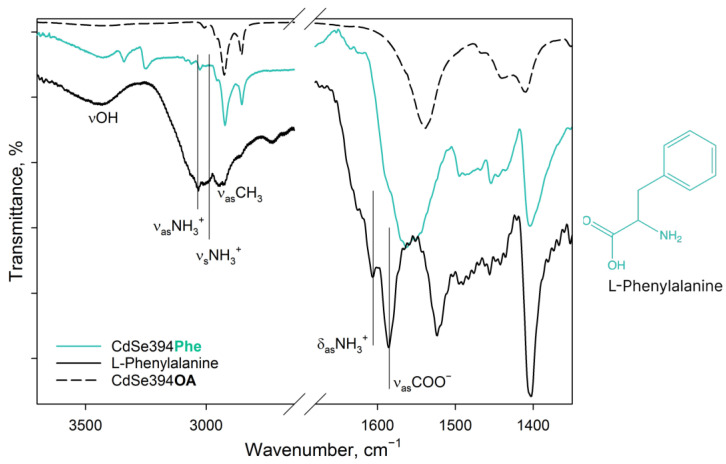
FTIR spectra of L-Phenylalanine-capped CdSe394Phe (turquoise line) sample. Black solid line shows the FTIR spectrum of free L-Phenylalanine reference. Dashed black line shows the FTIR spectrum of the initial oleic-acid-capped CdSe394OA sample. Positions of the main vibration bands are marked by black solid lines. The spectra are offset for clarity.

Figure 4 shows FTIR spectra for pure L-Phenylalanine, a sample coated with the L-Phenylalanine ligand, and an initial sample with oleic acid on the surface. In the spectrum of pure L-Phenylalanine, mainly, the same bands are observed as in the spectrum of the previously described L-Alanine. In the spectrum of pure L-Phenylalanine are pronounced bands: vibrations of the hydroxyl group (3443 cm^−1^), asymmetric (3068 cm^−1^) and symmetric (3034 cm^−1^) stretching vibrations of the charged NH_3_^+^ group of the zwitterion form [40,42], alkyl vibrations (2964–2940 cm^−1^), asymmetric (1608 cm^−1^) and symmetric (1525 cm^−1^) deformation vibrations of the NH_3_^+^ group, and asymmetric vibrations of the carboxylate group COO^−^ (1587 cm^−1^) of the zwitterion form. In addition, there is a small contribution of aromatic C=C stretching vibrations in the form of a shoulder at 1625 cm^−1^ [44].

In the case of the CdSe394Phe sample capped with the L-Phenylalanine ligand on the surface, the FTIR spectrum (Figure 4) gives, at first sight, the same bands of the hydroxyl group (3449 cm^−1^) and the NH_3_^+^ group (3035 cm^−1^) as for pure L-Phenylalanine; however, the latter are noticeably less pronounced, while distinct bands at 3349/3259 cm^−1^, attributed to the NH_2_ group, appear [42]. Thus, it can be concluded that L-Phenylalanine exists in the anionic form on the surface of nanoparticles [42] and there is no unbound free ligand. For asymmetric vibrations of the carboxyl group (1567 cm^−1^), a shift to the region of lower energies is observed, which also corresponds to the anionic form and indicates attachment to the surface. At the same time, it is worth noting the presence of unsubstituted oleic acid, from which the vibrations of the hydrocarbon chains and the contribution to the fluctuations of the carboxyl group are visible.

For convenience, all of the main vibration assignments from the FTIR spectra of L-Alanine, CdSe394Ala, L-Phenylalanine, and CdSe394Phe shown in Figure 3 and Figure 4 are presented in Table 1.

### 3.3. Analysis of Optical and Chiroptical Properties

**Figure 5:**
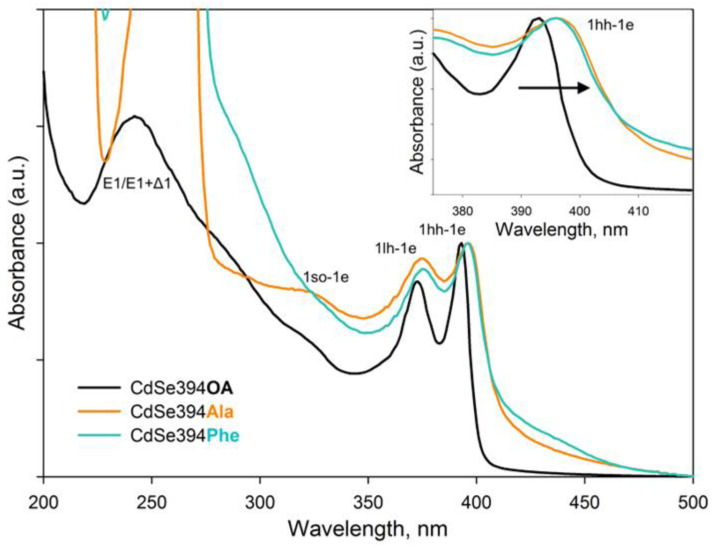
Typical absorbance spectra of CdSe394OA NPLs (black solid line) and their modification after ligand exchange with L-alanine (CdSe394Ala, orange line) and L-phenylalanine (CdSe394Phe, turquoise line) ligands. Black arrow in the insert shows spectral shift after ligand exchange.

The optical properties of nanoparticles coated with amino acids were analyzed using absorption and luminescence spectroscopy. The obtained absorption spectra are shown in Figure 5. For the initial CdSe394OA NPLs, standard exciton transitions from the zones of heavy holes (hh), light holes (lh), and spin-orbital (so) holes to the conduction band at wavelengths of 394, 366, and 320 nm, respectively, as previously noted in the literature [26,45], were observed. The wide absorption band at 240 nm refers to the transitions at the boundary of the 2D Brillouin zones (L point) [46].

The absorption spectra of CdSe NPLs coated with L-alanine and L-phenylalanine ligands are similar to each other and retain exciton transitions of the initial sample, but the positions of these bands are shifted to the long-wavelength region. This shift may be in favor of the successful exchange of ligands on the surface and the resulting deformation of the crystal structure. As described earlier, CdSe NPLs collapse due to the effect of spontaneous folding in the case of deformations of the discrepancy between the volume of the ligand and the available seat on the surface of the nanoparticle [37]. Therefore, the replacement of the ligand itself inevitably leads to changes in the density of the surface coating and affects the electronic properties of such an atomically thin system. It also should be noted that it is not possible to distinguish the absorption band from the transitions at the border of the Brillouin zones since the absorption of organic ligands is superimposed.

**Figure 6:**
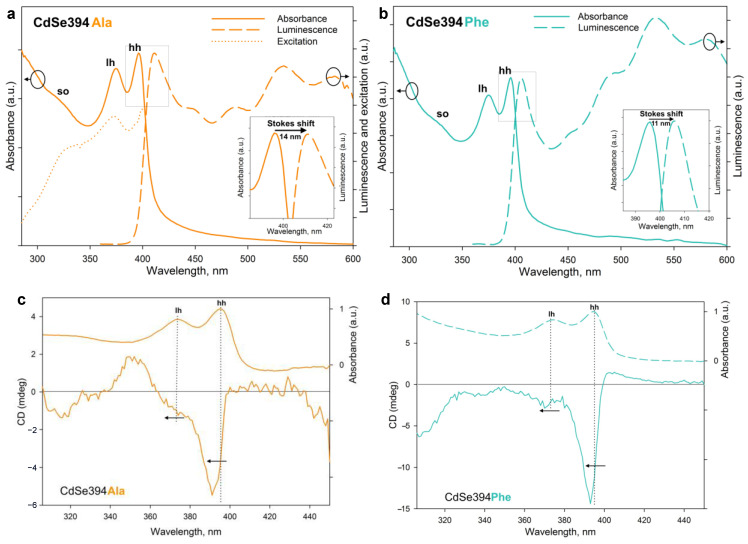
(**a**) Absorption, luminescence, and excitation spectra of CdSe394Ala (solid, dashed, and dotted orange lines, respectively). For luminescence spectrum, the excitation wavelength was 330 nm. The insert shows, in detail, the Stokes shift. (**b**) Absorption and luminescence spectra of CdSe394Phe (solid and dashed turquoise lines, respectively). For luminescence spectrum, the excitation wavelength was 330 nm. The insert shows, in detail, the Stokes shift. (**c**,**d**) CD spectra of CdSe394Ala and CdSe394Phe samples covered with L-Alanine (orange line) and L-Phenylalanine (turquoise line), respectively. Absorbance spectra are shown by dashed lines for comparison. Pointed lines show correspondence between CD and absorbance bands.

Figure 6a,b show the absorption, luminescence, and luminescence excitation spectra (for CdSe394Ala) of the samples obtained after ligand exchange. The luminescence excitation spectrum (Figure 6a) confirms the excitonic nature of the first luminescence band. The luminescence band corresponds to the lower exciton transition in terms of energy for both L-alanine (Figure 6a) and L-phenylalanine (Figure 6b); however, a bigger Stokes shift is observed for the former, probably from the broadening of the bands due to inhomogeneous surface coating with L-alanine. A wide absorption band at about 530 nm, also noticed for the initial CdSe394OA NPLs (Figure S4), is attributed to defective luminescence and was ascribed by [45] to non-passivated atoms on the surface. It can also be noted that for CdSe394Ala, the intensity of exciton luminescence is comparable to the defective one, whereas for CdSe394Phe, the latter prevails (this fact results in a turquoise luminescence of CdSe394Phe; see Figure S5).

Chiral ligands on the surface of the NPL are able to transfer their mirror asymmetry to the excitations in the semiconductor core NPL, thus inducing the chirality of the entire system. This phenomenon has been called chirality transfer and has been actively discussed recently [2,3,26,32]. The chiroptical properties of NPLs acquired in this way and the efficiency of chirality transfer in the case of chiral amino acid ligands were investigated using CD spectroscopy and demonstrated in Figure 6c,d. The typical CD spectrum for NPLs coated with the L-alanine ligand CdSe394Ala represents signs alternating bands in the UV-visible region from 300 to 450 nm, which confirms the influence of ligands on different interactions of excitons with right- and left-handed circularly polarized light. The position of the maxima corresponds with the transition bands in the absorption spectra and indicates the excitonic nature of CD transitions. Unlike the sulfur-containing ligands described earlier [26,32], CD bands corresponding to heavy and light holes (around 390 and 370 nm correspondingly) have the same sign, which is similar to the case described for zinc blende with an L-cysteine ligand on the surface [24,25]. However, a CD band corresponding to a spin-orbit hole with a positive sign at around 350 nm is visible only for the CdSe394Ala sample, possibly due to better passivation for the L-alanine ligand. In passing from L-alanine to L-phenylalanine, the signal intensity increases for CdSe394Phe, which is quite unexpected in view of a more complete substitution in the first case but may indicate the stronger effect of chirality induction for L-phenylalanine. The positions of the maxima are kept identical but shift in both cases from the position of exciton transitions in the absorption spectra.

The rotational strength of exciton transitions in CD spectra was analyzed using the g-factor of dissymmetry, given in Table 2, for the main exciton transitions for both CdSe394Ala and CdSe394Phe.

## 4. Conclusions

To summarize, we have studied the induction of chirality in ultimately thin CdSe nanoplatelets capped with enantiomers of thiol-free amino acids using the examples of L-alanine and L-phenylalanine, which resulted in chiral excitations in the 2D semiconductor core. We successfully grew colloidal atomically thin CdSe nanoplatelets of only a two monolayer thickness with lateral dimensions of the order of 100 nm and zinc blende structure, coated with native oleic acid ligands. To exchange the native oleic acid ligand for chiral thiol-free amino acids, we developed a new two-step approach using exchange for short-chain acetic acid followed by high-temperature exchange for amino acids with the distillation of acetic acid. According to FTIR analysis, both L-alanine and L-phenylalanine coordinate cadmium cations with the deprotonated carboxyl group on the basal Cd-rich (001) planes of nanoplatelets. The replacement of native oleic acid with amino acids preserves the electronic structure of heavy-hole, light-hole, and spin-orbit split-off excitons, and there is a slight red shift in the spectral position of all excitonic bands due to changes in mechanical stresses on the surface, as we assumed. After the exchange, the nanoplatelets retain bright excitonic luminescence, which is due to the absence of the quenching thiolate group in the ligands. Thiol-free amino acids successfully induce the pronounced chirality of exciton excitations in nanoplatelets, as shown using circular dichroism analysis, with a sufficiently high dissymmetry g-factor of up to –3.4 × 10^–3^ achieved for HH excitons in the case of L-phenylalanine. Thus, our work expands the variety of optically active ligands for chirality induction in cadmium chalcogenide nanoplatelets to the case of enantiomers of thiol-free compounds, and the developed native oleic acid ligand exchange method can be applied to a wide class of ligands with coordination through the carboxyl group.

## Figures and Tables

**Table 1:** Summary of essential vibration assignments from the FTIR spectra of L-Alanine, CdSe394Ala, L-Phenylalanine, and CdSe394Phe shown in Figure 3 and Figure 4.

Ala	CdSe394Ala	Phe	CdSe394Phe	Assignments [40,41,42,43,44]
3445	3438	3451	3449	νOH
3290	3290		3349/3259	νNH_2_
3087	3085	3040	3035	ν_as_NH_3_^+^
3000		3015		ν_s_NH_3_^+^
2944	2928	2964	2963	ν_as_CH_3_
2910	2858	2940	2925	ν_s_CH_3_
1621		1587	1567	ν_as_COO^−^
1587		1608		δ_as_NH_3_^+^
1516/1502		1525		δ_s_NH_3_^+^
1455				δ_as_CH_3_
1410				δ_s_CH_3_
1360				ν_s_COO^−^

**Table 2:** Spectral position λCD and dissymmetry g-factor for main circular dichroism bands of CdSe samples covered with L-alanine and L-phenylalanine ligands.

Sample	λ_CD_, nm	g-Factor (×10^−3^)
CdSe394Ala	395	−1.20
	374	−0.46
CdSe394Phe	395	−3.40
	374	−0.63

## Data Availability

The data that support the findings of this study are available from the corresponding author upon reasonable request.

## Supplementary Materials

The following supporting information can be downloaded at: https://www.mdpi.com/article/10.3390/ma17010237/s1, Figure S1: FTIR spectra of as-synthesized CdSe394OA (black solid line) and of intermediate exchanged CdSe394AcA (blue solid line). Figure S2: Low-magnification TEM image of CdSe394Phe NPLs covered with L-phenylalanine. Figure S3: The size distribution of NPLs (a) CdSe394Ala and (b) CdSe394Phe. Figure S4: Typical luminescence spectra of CdSe394OA NPLs (black solid line) and their modification after ligand exchange with Ala (CdSe394Ala, orange solid line) and Phe (CdSe394Phe, turquoise solid line) ligands. Figure S5: Photo of CdSe394Phe NPL sample (a) under ambient light and (b) luminescent under excitation of 370 nm.
