# Supplementary material for: Chirality in Atomically Thin CdSe Nanoplatelets Capped with Thiol-Free Amino Acid Ligands: Circular Dichroism vs. Carboxylate Group Coordination
Source: Materials (Basel). 2024 Jan 1;17(1):237. doi: 10.3390/ma17010237 (PMC10779562; doi:10.3390/ma17010237)
Supplement: Supplementary file 1 [file materials-17-00237-s001.zip › materials-2765810-supplementary.pdf]

Supporting Information:

Chirality in atomically thin CdSe nanoplatelets capped with thiol-free amino acid ligands: circular dichroism vs. carboxylate group coordination.

Daria A. Kurtina[1], *Vladimir B. Zaytsev*[2], *Roman B. Vasiliev*[1, 3 *]

[1] Department of Chemistry, Lomonosov Moscow State University, 119991, Moscow, Russia
[2] Department of Physics, Lomonosov Moscow State University, 119991, Moscow, Russia
[3] Department of Materials Science, Lomonosov Moscow State University, 119991, Moscow, Russia

Contents

Figure S1. FTIR spectra of as-synthesized CdSe394OA and of intermediate exchanged CdSe394AcA.

Figure S2. Low-magnification TEM images of CdSe394Phe NPLs covered with L-phenylalanine.

Figure S3: The size distribution of NPLs (a) CdSe394Ala and (b) CdSe394Phe.

Figure S4. Typical luminescence spectra of CdSe394OA NPLs and their modification after ligand exchange with L-Alanine (CdSe394Ala) and L-Phenylalanine (CdSe394Phe) ligands.

Figure S5. Photo of CdSe394Phe NPLs (a) under room light and (b) their luminescence under excitation of 370 nm.

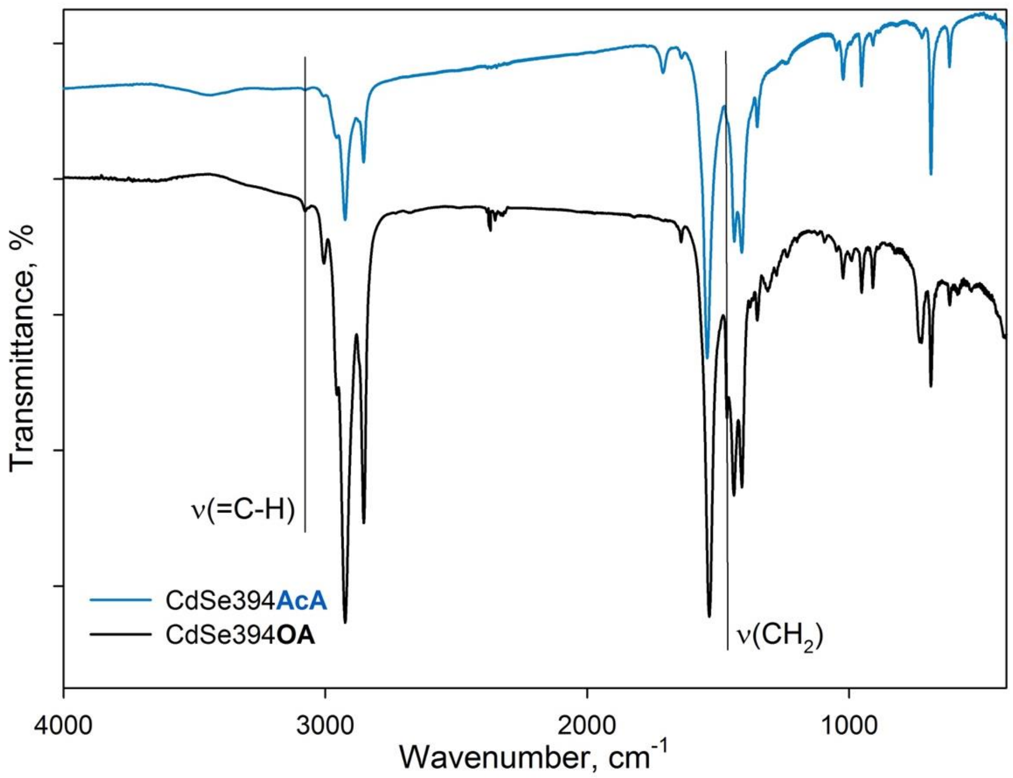

Figure S1. FTIR spectra of as-synthesized CdSe394OA (black solid line) and of intermediate exchanged CdSe394AcA (blue solid line).

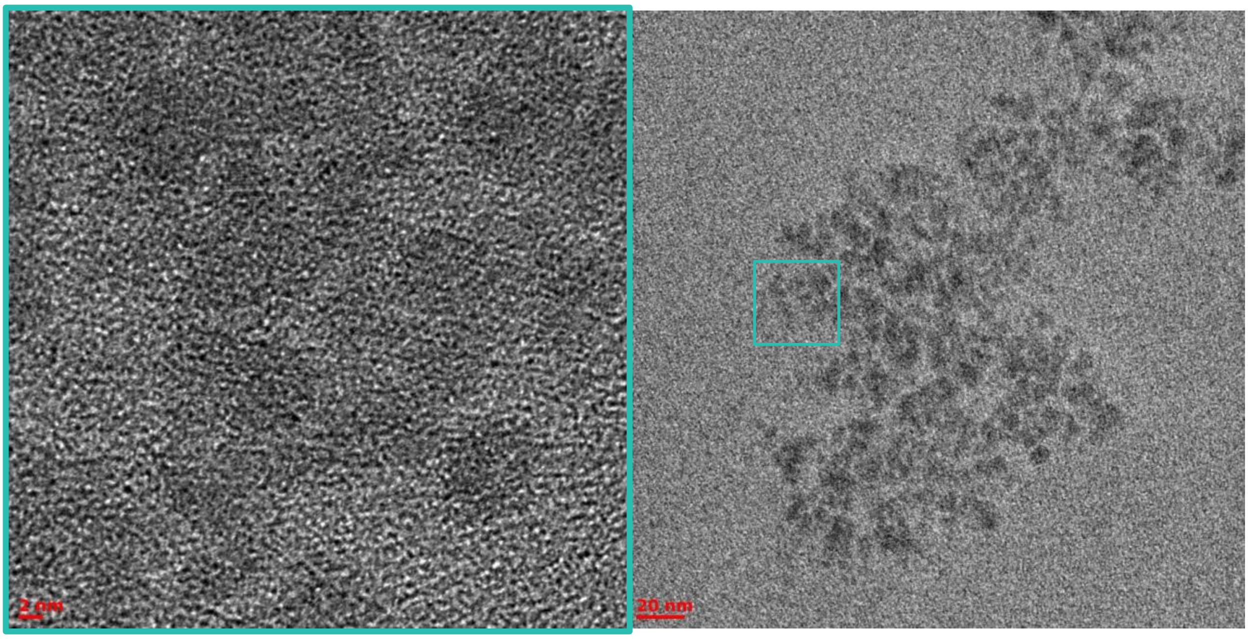

Figure S2. Low-magnification TEM images of CdSe394Phe NPLs covered with L-phenylalanine.

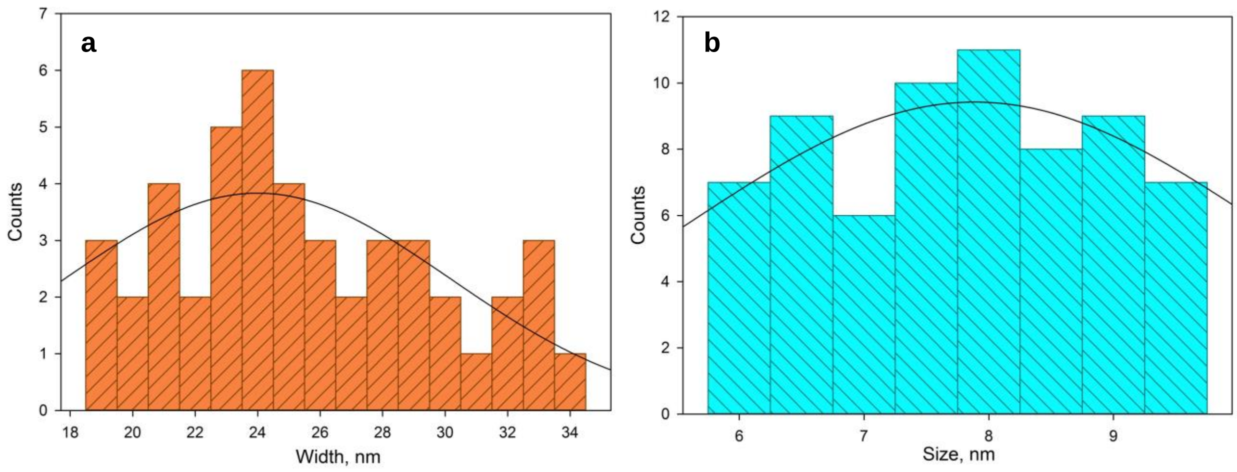

Figure S3: The size distribution of NPLs (a) CdSe394Ala and (b) CdSe394Phe.

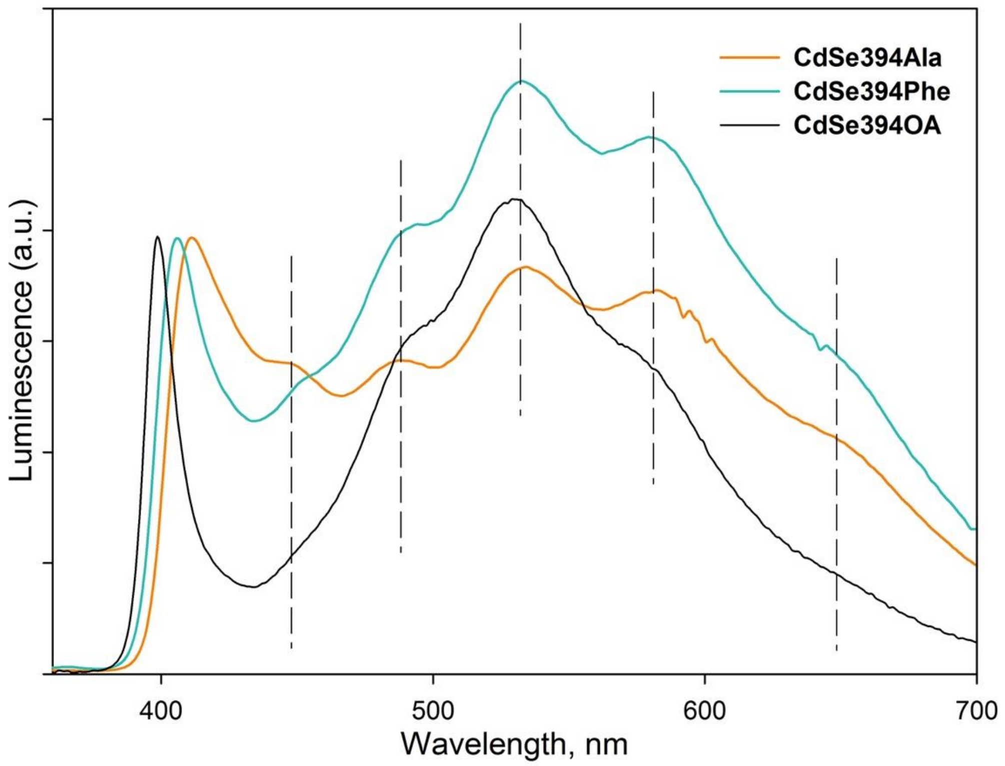

Figure S4. Typical luminescence spectra of CdSe394OA NPLs (black solid line) and their modification after ligand exchange with Ala (CdSe394Ala, orange solid line) and Phe (CdSe394Phe, turquoise solid line) ligands.

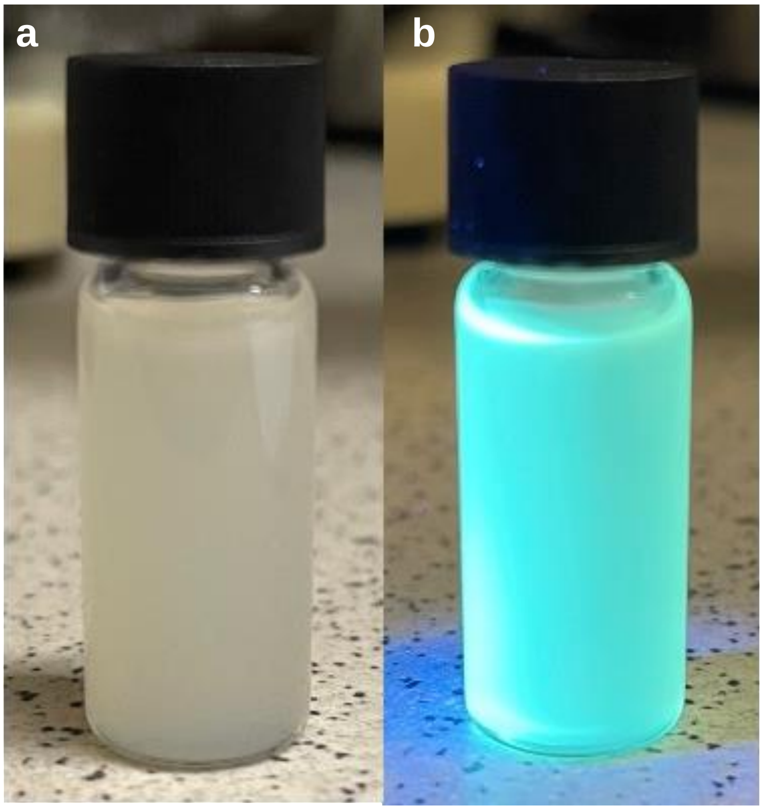

Figure S5. Photo of CdSe394Phe NPLs (a) under room light and (b) luminescent under excitation of 370 nm.
